# Comparative transcriptomics analysis identifies crucial genes and pathways during goose spleen development

**DOI:** 10.3389/fimmu.2024.1327166

**Published:** 2024-02-05

**Authors:** Shenqiang Hu, Yang Song, Xiaopeng Li, Qingliang Chen, Bincheng Tang, Jiasen Chen, Guang Yang, Haoyu Yan, Junqi Wang, Wanxia Wang, Jiwei Hu, Hua He, Liang Li, Jiwen Wang

**Affiliations:** ^1^ State Key Laboratory of Swine and Poultry Breeding Industry, College of Animal Science and Technology, Sichuan Agricultural University, Chengdu, China; ^2^ Key Laboratory of Livestock and Poultry Multi-Omics Ministry of Agriculture and Rural Affairs, College of Animal Science and Technology, Sichuan Agricultural University, Chengdu, China; ^3^ Animal Genetic Resources Exploration and Innovation Key Laboratory of Sichuan Province, College of Animal Science and Technology, Sichuan Agricultural University, Chengdu, China; ^4^ Department of Animal Production, General Station of Animal Husbandry of Sichuan Province, Chengdu, China

**Keywords:** Sichuan white goose, Landes goose, spleen, immunity, transcriptomic analysis, protein-protein interaction network

## Abstract

As the largest peripheral lymphoid organ in poultry, the spleen plays an essential role in regulating the body’s immune capacity. However, compared with chickens and ducks, information about the age- and breed-related changes in the goose spleen remains scarce. In this study, we systematically analyzed and compared the age-dependent changes in the morphological, histological, and transcriptomic characteristics between Landes goose (LG; *Anser anser*) and Sichuan White goose (SWG; *Anser cygnoides*). The results showed a gradual increase in the splenic weights for both LG and SWG until week 10, while their splenic organ indexes reached the peak at week 6. Meanwhile, the splenic histological indexes of both goose breeds continuously increased with age, reaching the highest levels at week 30. The red pulp (RP) area was significantly higher in SWG than in LG at week 0, while the splenic corpuscle (AL) diameter was significantly larger in LG than in SWG at week 30. At the transcriptomic level, a total of 1710 and 1266 differentially expressed genes (DEGs) between week 0 and week 30 were identified in spleens of LG and SWG, respectively. Meanwhile, a total of 911 and 808 DEGs in spleens between LG and SWG were identified at weeks 0 and 30, respectively. Both GO and KEGG enrichment analysis showed that the age-related DEGs of LG or SWG were dominantly enriched in the Cell cycle, TGF−beta signaling, and Wnt signaling pathways, while most of the breed-related DEGs were enriched in the Neuroactive ligand−receptor interaction, Cytokine−cytokine receptor interaction, ECM−receptor interaction, and metabolic pathways. Furthermore, through construction of protein-protein interaction networks using significant DEGs, it was inferred that three hub genes including *BUB1, BUB1B*, and *TTK* could play crucial roles in regulating age-dependent goose spleen development while *GRIA2*, *GRIA4*, and *RYR2* could be crucial for the breed-specific goose spleen development. These data provide novel insights into the splenic developmental differences between Chinese and European domestic geese, and the identified crucial pathways and genes are helpful for a better understanding of the mechanisms regulating goose immune functions.

## 1 Introduction

In avian species, the spleen, thymus, and bursa of Fabricius are the major immune organs, and they play important roles in regulation of both T and B lymphatic maturation and differentiation. Moreover, because of the relatively undeveloped lymphatic system, the spleen plays a more important role in the avian overall immune system compared to mammals (1). In addition to its hematopoietic and erythrocyte-clearing actions, the spleen is also the main site of specific immunity in the body (2). However, the avian immune organs gradually weaken with age and cease when reaching sexual maturity, followed by degeneration (3). It is generally believed that the spleen morphology can be used as an important indicator to evaluate the avian immunocompetence, and there is evidence that the avian spleen weight is affected by many factors including breed, age, season, and parasitic infections (1). Wu et al. showed that the native Beijing-you chickens are more resistant to disease than the imported White Leghorn chickens (4). It was observed that the spleen weight increases with age in White Pekin ducks (5), and a recent study also showed that adult geese were more resistant to pathogens than goslings because of more lymphocytes in their spleens (6). Oakeson (7) and Riddle (8) reported that the spleen weights of sparrows and pigeons significantly changed during the breeding cycle, being higher in spring and summer. Besides, the chicken spleen weight increased after being infected by parasites (9). Histologically, the avian spleen is mainly composed of the red pulp (RP), splenic corpuscle (AL), splenic trabecula (TL), trabecular artery (TA) and central artery (CA). The RP functions as a filter for blood and promotes the absorption of senescent red blood cells (10), and its special reticular structure serves as the basis of the blood-spleen barrier (11). The B lymphocytes are tightly distributed in the AL (12). The TL contains blood and lymph vessels as well as nerves, and lymphocytes can migrate through lymph vessels to the splenic lymph nodes (13). The TA and CA are where the blood circulation takes place (14). In this regard, the use of the spleen histology as a measurement of avian immunocompetence should be a more accurate way.

Multi-omics techniques have been frequently employed to investigate the regulatory mechanisms of mammalian immune organ development. However, compared with mammals, less studies have been conducted on avian immune organ development, especially for the regulatory mechanisms of goose immune organ development. Tariq et al. firstly *de novo* assembled the transcriptome of the goose peripheral blood lymphocytes and identified 125 important immune-related genes (15). Through comparative analysis of the spleen transcriptomes of gosling and adult geese, 22 immune-related genes, including immunoglobulin alpha heavy chain (*IgH*), mannan-binding lectin serine protease 1 isoform X1 (*MASP1*) and C–X–C chemokine receptor type 4 (*CXCR4*), were identified to be crucial for the higher susceptibility to pathogens in goslings than in adult geese (6). Also, melatonin was shown to be crucial for the avian spleen development and immunomodulation (16).

China is the leading goose producer in the world, and the goose industry plays an important role in Chinese agricultural economics. Both Landes goose (LG), which is one of European domestic goose breeds that originate from graylag geese (*Anser anser*), and Sichuan White goose (SWG), which is one of Chinese domestic goose breeds that originate from swan geese (*Anser cygnoides*), are widely distributed in most regions of China and frequently used in commercial goose production, due to their outstanding production performance. Considering the observations that the two goose breeds show different immune capacities and environmental adaptations, the present study aimed to compare the age-dependent changes in their splenic histomorphological and functional characteristics as well as the underlying regulatory mechanisms. These results are expected to provide novel insights into the molecular mechanisms regulating the postnatal spleen developmental dynamics and the development of immunological competence in geese.

## 2 Materials and methods

### 2.1 Animals and sample collection

All LG and SWG used in this study were reared at the Waterfowl Breeding Farm of Sichuan Agricultural University (Ya’an, Sichuan, China). A hundred 1-day-old male geese from each of these two goose breeds were hatched from the same batch. All geese were reared in brooders from 0-3 weeks of age and thereafter shifted to the net-floor mixed rearing systems, where the geese were reared in an indoor area with a size of length × width: 6 m × 13 m, consisting of a 60 m^2^ plastic net at a height of 1 m above the ground level and an 18 m^2^ fermentation bed. The lighting schedule is 16 hours on and 8 hours off, with lights on at 8:00 am. At 0, 6, 10, and 30 weeks of age, there were 8 male geese of similar body weights randomly selected from each breed for sample collection. At each sampling time point, the goose live body weights were firstly weighted after 12 hours of fasting and then slaughtered for determination of the spleen and thymus weights (n = 8 per breed per week). The organ indexes of spleen and thymus were calculated using the following formula: organ index (‰) = (organ weight (g)/body weight (g)) × 1000‰. For each goose breed, the spleens from 4 individuals at each sampling time point (n = 4 per breed per week) were used for histological examination, and the spleens from the remaining slaughtered geese were rapidly frozen with liquid nitrogen and stored at -80°C until RNA extraction.

### 2.2 Histological observation

The spleen tissue was fixed in 4% paraformaldehyde at room temperature for over 72 hours, dehydrated in a series of graded ethanol, transferred to xylene, and embedded in paraffin wax. Subsequently, each sample was cut into 5-µm thick slices for hematoxylin and eosin (H&E) staining. Finally, the stained sections were placed under a microscope (BX53, OLYMPUS, America) for observation and photography. The splenic histological parameters, including the RP area, AL diameter, TL area, TA diameter, and CA diameter, were analyzed using Image-Pro Plus 6.0 software (National Institutes of Health, Bethesda, MD).

### 2.3 RNA extraction and quality assessment

Total RNA used for transcriptomic sequencing and real‐time reverse transcription‐quantitative polymerase chain reaction (RT-qPCR) analysis were extracted from the spleens collected from these two goose breeds at 0 and 30 weeks of age (n=3 per breed per week) by the RNAiso Plus kit (Vazyme, Nanjing, China) following the manufacturer’s instruction. The RNA concentration and integrity was determined using a NanoDrop 2000 Micro-ultraviolet Spectrophotometer (Thermo Fisher Scientific, Wilmington, NC) and Agilent 2100 Bioanalyzer (Agilent Technologies, Santa Clara, CA), respectively. According to the manufacturer’s instructions, the RNA-seq libraries were prepared using the Illumina TruSeq mRNA Sample Preparation Kit (Illumina, San Diego, CA, USA) and sequenced on the Illumina Hiseq X-Ten platform.

### 2.4 Transcriptomic bioinformatics analysis

The quality control of our transcriptomic sequencing data was performed using both FastQC (version 0.11.9) and fastp (version 0.22.0) software (17) to obtain the clean reads. The adaptor reads, poly-N contained reads (N% > 10%), and low-quality reads were removed, and the clean reads were mapped to our recently assembled reference-grade goose genome by Hisat2 (version 2.0.5). The SAMtools (version 1.6.0) software was used to convert and sort the SAM files to binary alignment/mapping (BAM) files (18). The StringTie (version 2.2.1) software was used to calculate the expression levels of each transcript (19). Subsequently, the differentially expressed genes (DEGs) between LG at 0 week of age (LG0), LG at 30 weeks of age (LG30), SWG at 0 week of age (SWG0), and SWG at 30 weeks of age (SWG30) were identified using DESeq2 (version 1.34.0) package, with the screening criteria of |log_2_Foldchange| > 1 and *P* value < 0.05 (20). Both Gene Ontology (GO) and Kyoto Encyclopedia of Genes and Genomes (KEGG) functional enrichment analysis were performed using KOBAS (version 3.0) software (21). The relationships of DEGs were identified by STRING 10 database, and the Cytoscape (version 3.7.1) software was used to visualize the interaction networks (22). The key genes in the protein-protein interaction (PPI) networks were further identified using the molecular complex detection (MCODE) and CytoHubba plugin of Cytoscape software (23, 24).

### 2.5 RT-qPCR analysis

The RT-qPCR assay was used to validate the expression patterns of some selected DEGs identified by our transcriptomic sequencing. Equal amounts of total RNA extracted from each sample were reversely transcribed into the cDNAs using the HiScript III RT SuperMix for RT-qPCR kit (+gDNA wiper) (Vazyme, Nanjing, China). The RT-qPCR primer pairs of these selected genes were designed using the Primer Premier 5.0 software and are listed in **Supplementary Table 1**. Reactions of RT-qPCR were performed on the Bio-Rad CFX96 real-time PCR detection system (Bio-Rad, Hercules, CA, USA). Each reaction was performed in a 20 μL volume, containing 10 μL 2 × ChamQ SYBR qPCR Master Mix (Vazyme, Nanjing, China), 0.4 μL of each forward and reverse primer, 2 μL cDNA, 7.2 μL ddH_2_O. The RT-qPCR amplification conditions were listed as follows: pre-denaturation at 95°C for 30 s; followed by 40 cycles of 95°C for 10 s and 60°C for 30 s. Each sample was run in triplicate. The relative mRNA expression levels of these DEGs were normalized using the two reference genes *GAPDH* and *β-ACTIN* according to the comparative Ct method (25).

### 2.6 Statistical analysis

The splenic morphological and histological data were firstly analyzed using Excel software, and the results were expressed as the mean ± SEM. Subsequently, data were analyzed by two-way analysis of variance (ANOVA) using the general linear model (GLM) procedure of statistical analysis software (SAS) 9.4 software, with age and breed as the fixed factors. When a significant effect was observed, the *post-hoc* Duncan’s multiple range test was used to assess significant differences between different weeks of age or breeds. A probability (*P*) value less than 0.01 was considered statistically extremely significant different, while a *P* value less than 0.05 was considered significant different. Finally, both GraphPad Prism 8.0 and R 4.2.1 software were used to visualize these results.

## 3 Results

### 3.1 Comparison of the age-dependent morphological changes in the immune organs between LG and SWG

**Figure 1:**
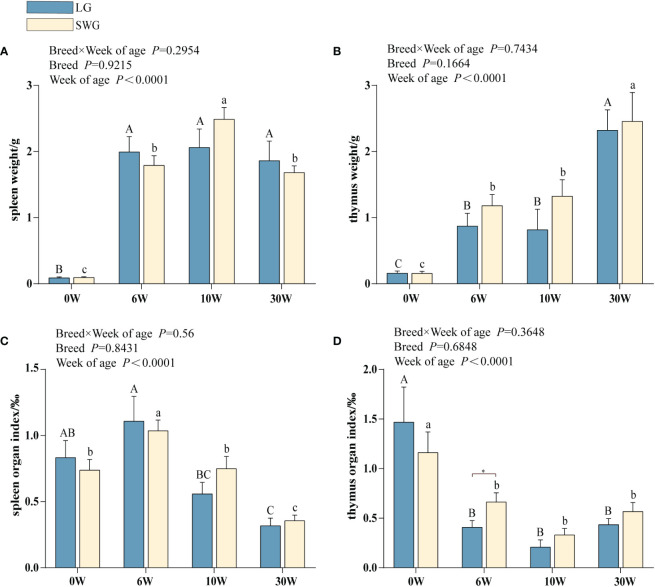
Developmental changes in the morphology of the immune organs between LG and SWG. **(A)** Spleen weight. **(B)** Spleen organ index. **(C)** Thymus weight. **(D)** Thymus organ index. “*” indicates significant differences between the two designated groups at the level of *P* < 0.05. Different capital letters indicate significant differences across weeks in LG at *P* < 0.05. Different lowercase letters indicate significant differences across weeks in SWG at *P* < 0.05. LG, Landes goose; SWG, Sichuan White goose; W, weeks of age.

As depicted in **Figures 1A, B**, the weights of spleen and thymus increased significantly with age in both LG and SWG (*P* < 0.05). The spleen weights reached the peak at 10 weeks of age in both LG and SWG, and the spleen weight of SWG was significantly higher at week 10 than at the other weeks (*P* < 0.05). At 30 weeks of age, the spleen weights of both goose breeds declined, and LG had slightly higher spleen weights compared to SWG (*P* > 0.05). The splenic organ index reached the peak at 6 weeks of age (*P* < 0.05) in both LG and SWG, followed by a gradual decline (**Figure 1C**). The thymus weights reached the peak at 30 weeks of age (*P* < 0.05) in both LG and SWG, and the thymus organ index exhibited a distinct pattern from its weight, showing a declining trend in both LG and SWG. At 6 weeks of age, the thymus organ index was significantly higher in SWG than in LG (*P* < 0.05) (**Figure 1D**).

### 3.2 Comparison of the age-dependent histological changes in the spleen between LG and SWG

**Figure 2:**
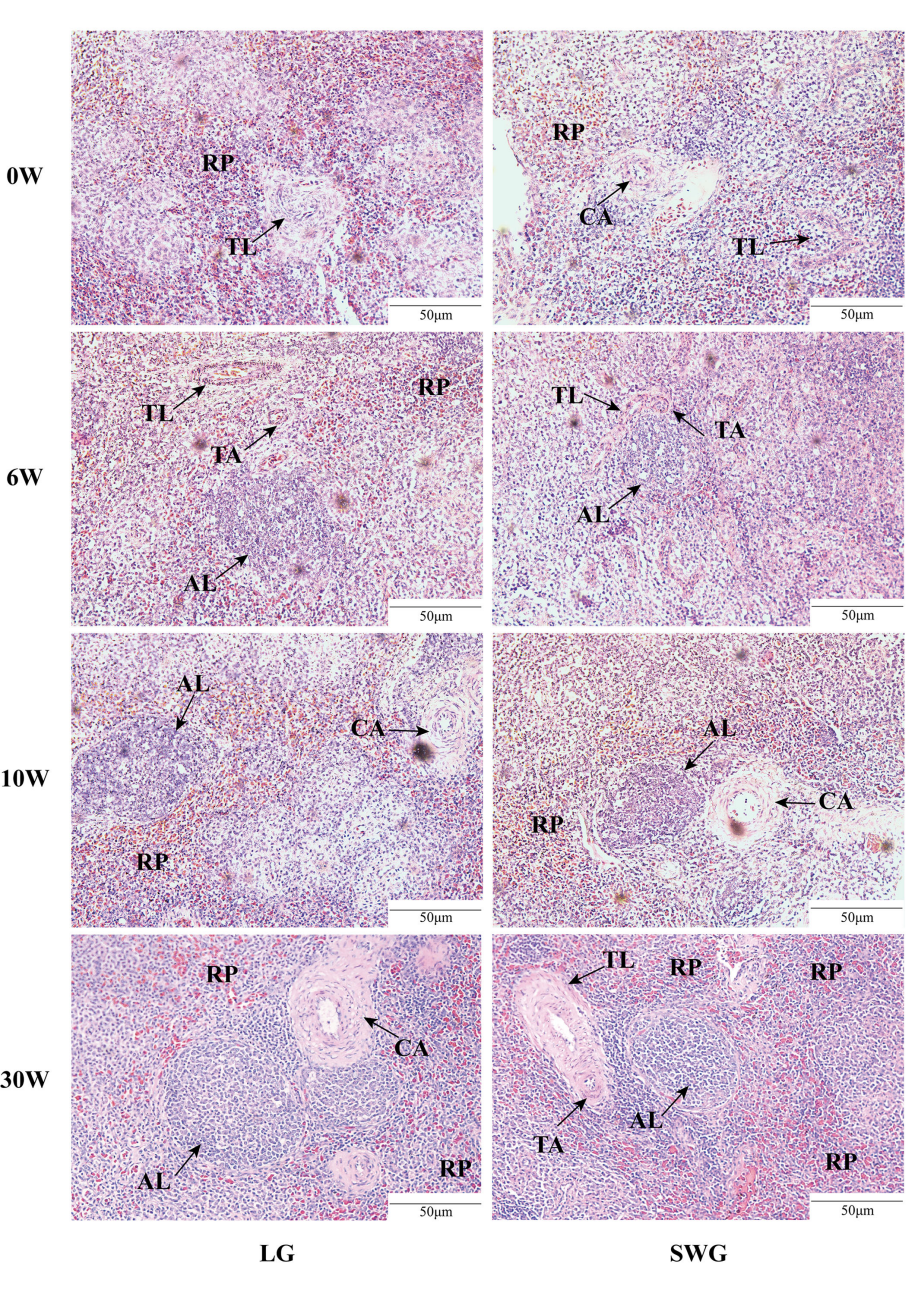
Histological observation of the spleens from LG and SWG at different weeks of age. Representative histological images (200×) of the spleens from LG and SWG at 0, 6, 10 and 30 weeks of age. RP, red pulp; AL, splenic corpuscle; TL, splenic trabecula; TA, trabecular artery; CA, central artery; LG, Landes goose; SWG, Sichuan White goose; W, weeks of age.

**Figure 3:**
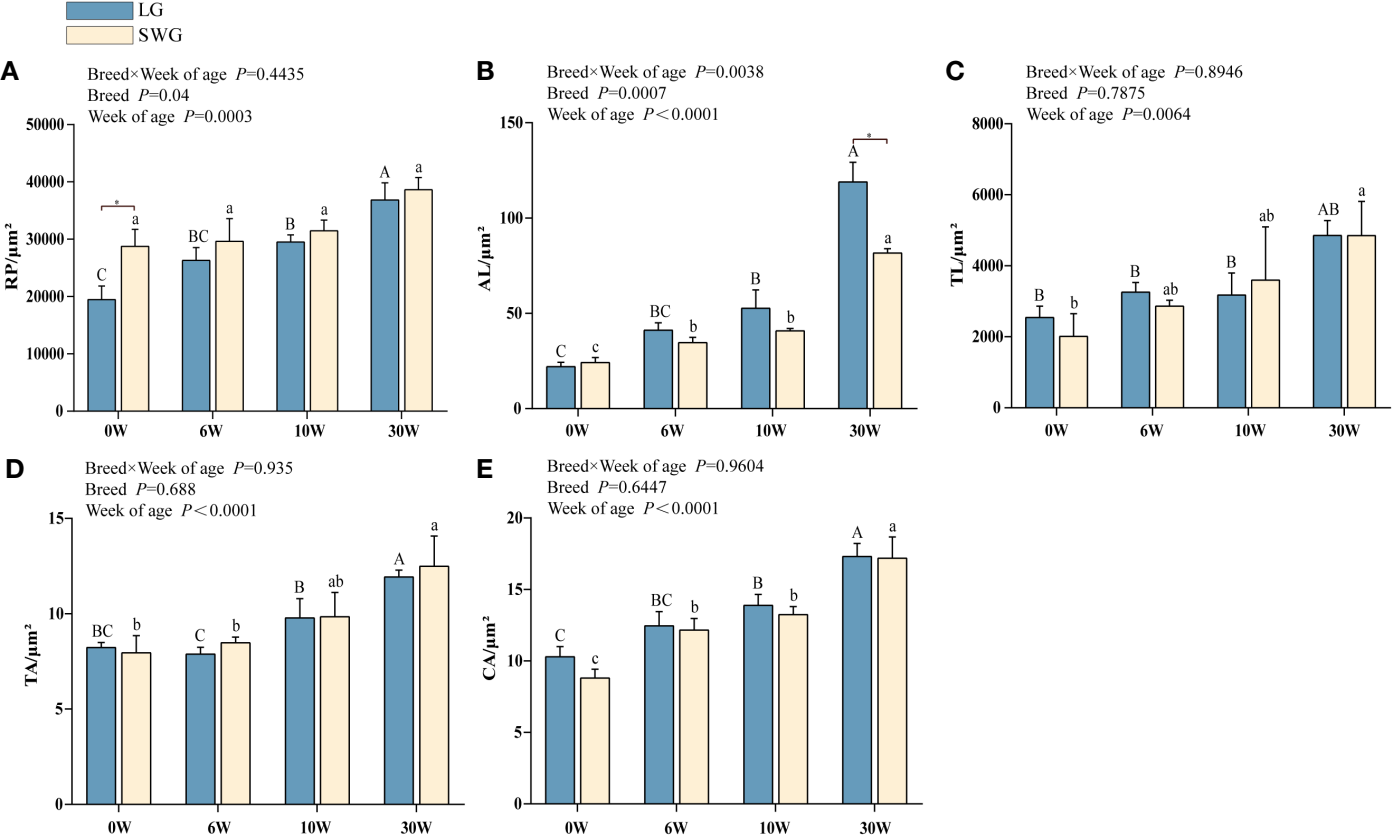
Developmental changes in the spleen histological characteristics between LG and SWG. **(A)** Red pulp area. **(B)** Splenic corpuscle diameter. **(C)** Splenic trabeculae area. **(D)** Trabecular artery diameter. **(E)** Central artery diameter. “*” indicates significant differences between the two designated groups at *P* < 0.05. Different capital letters indicate significant differences across weeks in LG at *P* < 0.05. Different lowercase letters indicate significant differences across weeks in SWG at *P* < 0.05. RP, red pulp; AL, splenic corpuscle; TL, splenic trabeculae; TA, trabecular artery; CA, central artery; LG, Landes geese; SWG, Sichuan White geese; W, weeks of age.

Next, the age-dependent spleen histological changes were analyzed and compared between LG and SWG. As shown in **Figure 2**, at 0-6 weeks of age, the RP area continuously increased, the periarterial lymphatic sheath was formed, and the AL appeared, but the boundary was not clear. At 10 weeks of age, the AL was intact and clear, and the lymphocytes around the CA increased. At 30 weeks of age, the splenic histological structure was more clearly visible, which represented the characteristic of a mature goose spleen. We further analyzed the differences in the spleen histological parameters between LG and SWG. As depicted in **Figure 3**, the RP area, AL diameter, TL area, TA diameter, and CA diameter significant increased with age in both LG and SWG (*P* < 0.05). Notably, at 0 week of age, SWG exhibited a significantly higher RP area compared to LG (*P* < 0.05) (**Figure 3A**). At 30 weeks of age, the AL diameter was significantly higher in LG than in SWG (*P* < 0.05) (**Figure 3B**).

### 3.3 Comparison of the age- and breed-related transcriptomic changes in the spleen between LG and SWG

To explore the molecular mechanisms regulating the goose spleen development, the spleens from both two goose breeds at 0 and 30 weeks of age were used for transcriptomic analysis. A total of 280,512,944 raw reads were obtained from these 12 samples, and an average of 23,373,275 clean reads were obtained from each sample after strict quality control. The mapping rate of all samples ranged from 90.00% to 93.38% (**Supplementary Table 2**). Results from both principal component analysis (PCA) and hierarchical clustering heatmap showed that these samples were clustered together according to the goose breed and developmental stage (**Supplementary Figure 1**). These data collectively demonstrated the high quality and biological repeatability of our sequencing data, which can be used for subsequent bioinformatic analysis.

**Figure 4:**
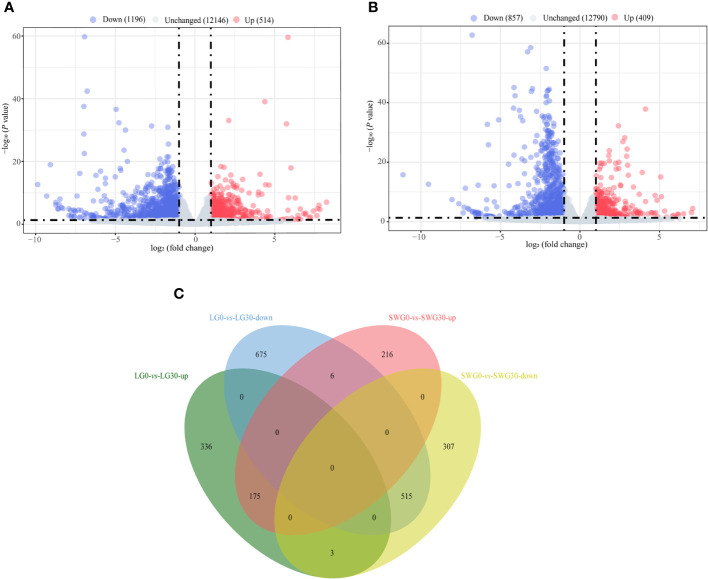
The age-dependent transcriptomic changes in spleens of LG and SWG. **(A)** Volcano diagram of the spleen DEGs between LG0 and LG30. **(B)** Volcano diagram of the spleen DEGs between SWG0 and SWG30. **(C)** Venn diagram of the spleen DEGs between LG and SWG. LG0, Landes goose at 0 week of age; LG30, Landes goose at 30 weeks of age; SWG0, Sichuan White goose at 0 week of age; SWG30, Sichuan White goose at 30 weeks of age; up, upregulated DEGs; down, downregulated DEGs.

**Figure 5:**
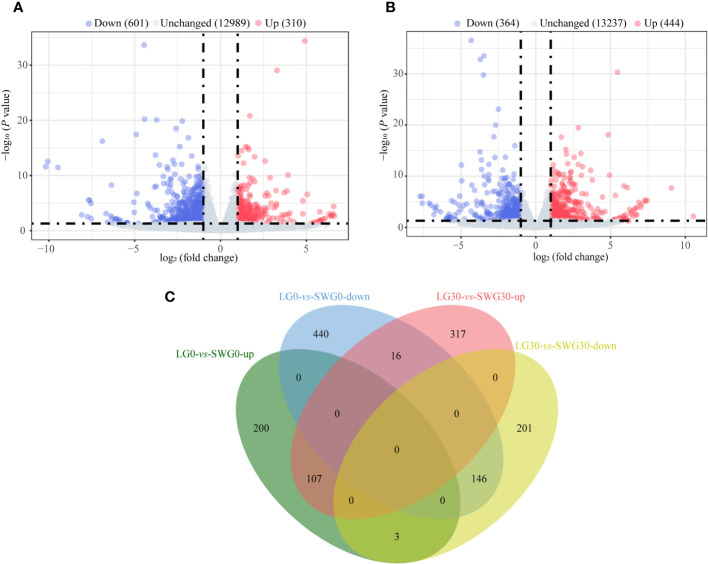
The breed-related transcriptomic changes in the goose spleen at different weeks of age. **(A)** Volcano diagram of the spleen DEGs between LG0 and SWG0. **(B)** Volcano diagram of the spleen DEGs between LG30 and SWG30. **(C)** Venn diagram of the spleen DEGs between LG and SWG. LG0, Landes goose at 0 week of age; LG30, Landes goose at 30 weeks of age; SWG0, Sichuan White goose at 0 week of age; SWG30, Sichuan White goose at 30 weeks of age; up, upregulated genes; and down, downregulated genes.

By comparing the splenic transcriptomes of each goose breed between different weeks of age, a total of 1,710 DEGs, including 514 upregulated- and 1,196 downregulated DEGs, were identified in spleens of LG between 0 and 30 weeks of age (**Figure 4A**). A total of 1,266 DEGs, including 409 upregulated- and 857 downregulated DEGs, were identified in spleens of SWG between 0 and 30 weeks of age (**Figure 4B**). The Venn diagram identified 175 commonly upregulated- and 515 commonly downregulated DEGs during the period from 0 to 30 weeks of age in both LG and SWG, while there were 9 age-related DEGs showing the opposite expression patterns between LG and SWG (**Figure 4C**). By comparing the splenic transcriptomes of LG and SWG at the same week of age, a total of 911 DEGs, including 310 upregulated- and 601 downregulated DEGs, were identified in spleens between LG and SWG at 0 week of age (**Figure 5A**). A total of 808 DEGs, including 444 upregulated- and 364 downregulated DEGs, were identified in spleens between LG and SWG at 30 weeks of age (**Figure 5B**). The Venn diagram identified 107 commonly upregulated- and 146 commonly downregulated DEGs between LG and SWG at both 0 and 30 weeks of age, while there were 19 breed-related DEGs showing the opposite expression patterns between these two weeks (**Figure 5C**).

### 3.4 Functional enrichment analysis of the age- and breed-related DEGs

**Figure 6:**
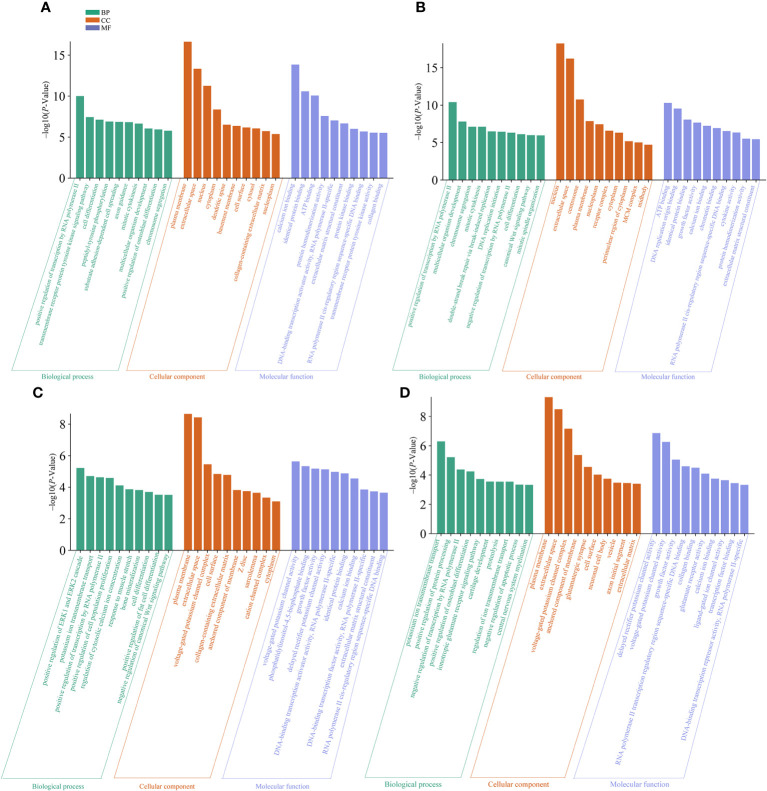
The top 10 significantly enriched GO terms by the age- and breed-related DEGs. **(A)** GO terms enriched by the spleen DEGs between LG0 and LG30. **(B)** GO terms enriched by the spleen DEGs between SWG0 and SWG30. **(C)** GO terms enriched by the spleen DEGs between LG0 and SWG0. **(D)** GO terms enriched by the spleen DEGs between LG30 and SWG30.

We performed GO enrichment analysis for the DEGs identified in spleens of each goose breed between different weeks of age. For LG, the top 30 of 2,409 GO terms were presented in **Figure 6A**. These DEGs were found to be mostly enriched in plasma membrane, extracellular space, nucleus, calcium ion biding, and positive regulation of transcription by RNA polymerase II. For SWG, the top 30 of 2,030 GO terms were presented in **Figure 6B**. These DEGs were found to be mostly enriched in nucleus, extracellular space, centrosome, ATP binding, and positive regulation of transcription by RNA polymerase II. Then, we performed GO enrichment analysis for the DEGs identified in spleens of the same age between LG and SWG. At 0 week of age, the top 30 of 1,658 GO terms were presented in **Figure 6C**. These DEGs were mostly enriched in plasma membrane, extracellular space, voltage-gated potassium channel activity, and positive regulation of ERK1 and ERK2 cascade. At 30 weeks of age, the top 30 of 1,397 GO terms were presented in **Figure 6D**. These DEGs were mostly enriched in plasma membrane, extracellular space, delayed rectifier potassium channel activity, and potassium ion transmembrane transport. Taken together, the DEGs identified in spleens of each goose breed between different weeks of age were mostly enriched in the GO terms related to growth and metabolism, while those in the spleen of the same age between the two goose breeds were mostly enriched in the GO terms related to protein synthesis.

**Figure 7:**
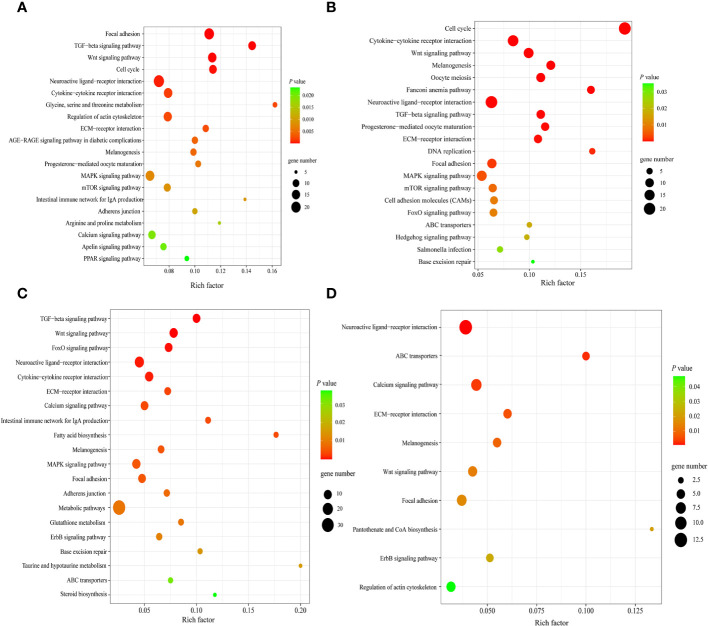
The top significantly enriched KEGG pathways by the age- and breed-related DEGs. **(A)** Top 20 significantly enriched KEGG pathways by the spleen DEGs between LG0 and LG30. **(B)** Top 20 significantly enriched KEGG pathways by the spleen DEGs between SWG0 and SWG30. **(C)** Top 20 significantly enriched KEGG pathways by the spleen DEGs between LG0 and SWG0. **(D)** Top 10 significantly enriched KEGG pathways by the spleen DEGs between LG30 and SWG30.

Next, KEGG enrichment analysis was performed on DEGs in spleens of each goose breed between different weeks of age. For LG, the top 20 of 110 KEGG pathways were presented in **Figure 7A**. These DEGs were found to be significantly enriched in Focal adhesion, TGF−beta signaling, Wnt signaling, Cell cycle, and Neuroactive ligand minus;receptor interaction pathways. For SWG, the top 20 of 100 KEGG pathways were presented in **Figure 7B**. These DEGs were significantly enriched in Cell cycle, Cytokine−cytokine receptor interaction, Wnt signaling, and Neuroactive ligand−receptor interaction pathways. Then, we performed KEGG enrichment analysis for the DEGs identified in spleens of the same age between LG and SWG. At 0 week of age, the top 20 of 96 KEGG pathways were presented in **Figure 7C**. These DEGs were significantly enriched in TGF−beta signaling, Neuroactive ligand−receptor interaction, Metabolic pathways, Cytokine−cytokine receptor interaction, and ECM−receptor interaction pathways. At 30 weeks of age, the top 10 of 75 KEGG pathways were presented in **Figure 7D**. The DEGs were significantly enriched in Neuroactive ligand−receptor interaction, Calcium signaling, and ECM−receptor interaction pathways.

### 3.5 PPI analysis of the age- and breed-related DEGs

**Figure 8:**
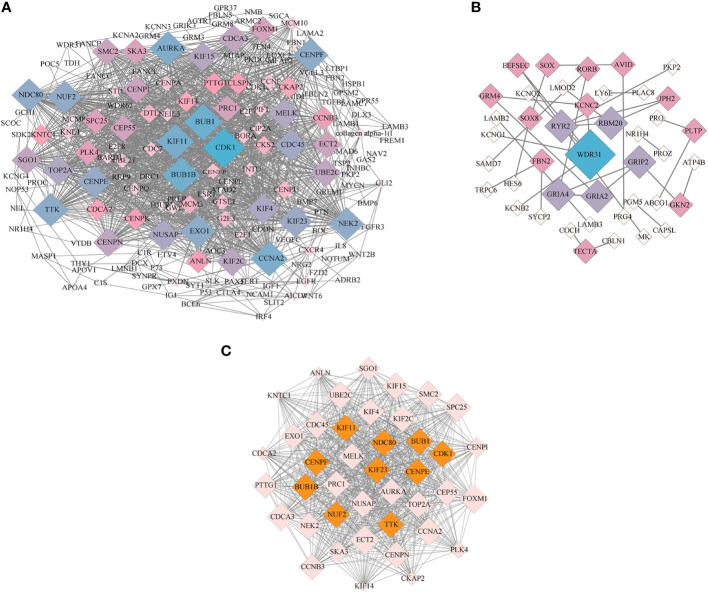
PPI network and MCODE analysis. **(A)** The PPI network constructed with the age-related DEGs in spleens of LG and SWG. **(B)** The PPI network constructed with the breed-related DEGs in spleens between LG and SWG. **(C)** The key module screened from the PPI network shown in **(A)** The top10 hub genes screened by the CytoHubba plugin were filled in orange. The size (area) of each node was drawn proportional to its degree of interaction, and the color of each node indicated its betweenness centrality. The value of degree represents the number of nodes directly connected to this node.

To further identify the key genes and pathways during goose spleen development, we performed PPI network analysis for the 690 commonly up- and downregulated (age-related) DEGs identified in spleens of LG and SWG between different weeks of age (**Figure 8A**) and the 253 commonly up- and downregulated (breed-related) DEGs identified in spleens between LG and SWG at both 0 and 30 weeks of age (**Figure 8B**), respectively. Moreover, we screened an important module containing 41 DEGs from the PPI network constructed with the 690 age-related DEGs using the MCODE plugin in Cytoscape (**Figure 8C**). The top 10 hub genes were also identified from the 690 age-related DEGs using the Cytohubba plugin, and all of them were functionally annotated to be involved in the above-screened module (**Table 1**). Based on both GO and KEGG enrichment analysis, we found that there were 4 hub genes significantly enriched in the Cell cycle pathway, including cyclin-dependent kinase 1 (*CDK1*), budding uninhibited by benzimidazoles 1 (*BUB1*), *BUB1* mitotic checkpoint serine/threonine kinase B (*BUB1B*), and *TTK* protein kinase (*TTK*), and there were 3 hub genes significantly enriched in the GO-BP term ‘cell division’, including *CDK1*, *NUF2* component of *NDC80* kinetochore complex (*NUF2*), and *NDC80* kinetochore complex component (*NDC80*). Besides, we also identified the top 10 hub genes from the 253 breed-related DEGs. As shown in **Supplementary Table 3**, there were 3 hub genes significantly enriched in the Neuroactive ligand-receptor interaction pathway, including glutamate ionotropic receptor AMPA type subunit 2 (*GRIA2*), glutamate ionotropic receptor AMPA type subunit 4 (*GRIA4*), and glutamate metabotropic receptor 4 (*GMR4*), and there were 4 hub genes significantly enriched in the GO-CC term ‘plasma membrane’, including *GRIA2*, *GRIA4*, ryanodine receptor 2 (*RYR2*), and tectorin alpha (*TECTA*). Also, the hub gene fibrillin 2 (*FBN2*) was observed to be significantly enriched in the Calcium ion binding pathway.

**Table 1:** Top 10 hub genes identified in PPI network constructed with the age-related DEGs.

Gene	Gene name	Degree	KEGG pathway/GO term
*CDK1*	Cyclin-dependent kinase 1	68	Cell cycle/Cell division
*BUB1*	Budding uninhibited by benzimidazoles 1	62	Cell cycle
*BUB1B*	*BUB1* mitotic checkpoint serine/threonine kinase B	61	Cell cycle
*KIF11*	Kinesin family member 11	60	Nucleus
*CENPF*	Centromere protein F	56	Nucleoplasm
*TTK*	*TTK* protein kinase	56	Cell cycle/Nucleus
*NUF2*	*NUF2* component of *NDC80* kinetochore complex	55	Cell division
*NDC80*	*NDC80* kinetochore complex component	55	Cell division
*CENPE*	Centromere protein E	54	Nucleoplasm
*KIF23*	Kinesin family member 23	53	Nucleus

Degree, the number of nodes directly connected to this node.

### 3.6 Validation of the expression patterns of several DEGs by RT-qPCR

**Figure 9:**
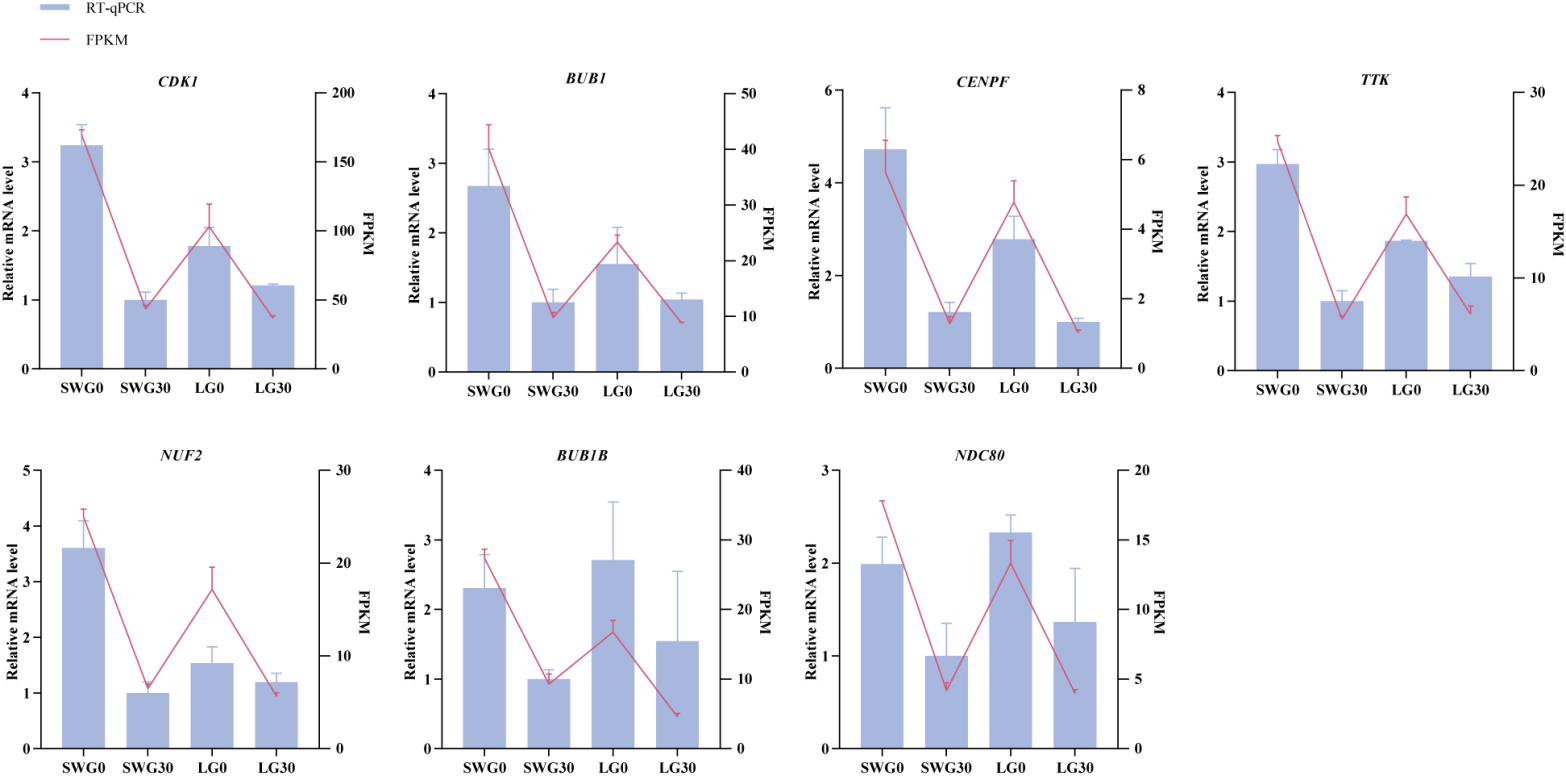
RT-qPCR validation of the expression patterns of seven DEGs.

To evaluate the accuracy of our transcriptomic sequencing data, seven DEGs (*CDK1*, *BUB1*, *CENPF*, *TTK*, *NUF2*, *BUB1B*, and *DNC80*) that were identified through PPI analysis were selected for RT-qPCR validation. As shown in **Figure 9**, the expression profiles of these DEGs determined by RT-qPCR showed similar trends to those observed by RNA-Seq, supporting the reliability of our transcriptomic sequencing results.

## 4 Discussion

The spleen, known as the largest lymphatic organ in poultry, plays important roles in regulating the body’s immunity and hematopoiesis, with the blood-spleen barrier being essential for resisting various pathogens (26). During poultry embryonic development, the spleen becomes a secondary lymphoid organ, providing an indispensable microenvironment for the interactions between lymphocytes and non-lymphocytes. Due to the underdeveloped lymphatic vessels and lymph nodes in poultry, the contribution of spleen to the poultry entire immune system can be more important than the mammalian counterpart (27). If the spleen is not well-developed, there will lack enough mature lymphocytes to resist pathogens (13). In the present study, we systematically analyzed and compared the age-dependent histomorphological and transcriptomic changes in spleens of two economically important goose breeds to reveal the molecular mechanisms underlying the goose postnatal spleen development.

Morphological results showed that the spleen weights of both goose breeds reached the peak at 10 weeks of age and then declined, while their thymus weights showed a continuous increasing trend. Meanwhile, we found that the spleen organ index reached the peak at 6 weeks of age and then declined. These above results indicated that the goose spleen develops most rapidly during the period from 0 to 10 weeks of age. It is commonly believed that weight loss is linked to the degeneration of spleen. However, this is not always the case, because the spleen histological index can better reflect its developmental potential (1). Histologically, it was observed that all examined histological indexes of the spleens from two goose breeds displayed an increasing trend with age, reaching the highest levels at 30 weeks of age. Previous studies have shown that the number of macrophages in the RP gradually increases during development, enabling a stronger immunity (28), and the resistance of the blood-spleen barrier to disease increases with the enlargement of the RP area (26). At 0 week of age, the RP area was significantly higher in SWG than in LG, implying that the SWG might be more resistant to disease than LG due to more macrophages in the RP. It has been reported that the B lymphocytes in the AL are required for the initiation of T lymphocytes-mediated immune response, and the abnormal development of B lymphocytes can result in immunodeficiency (12, 29). At 30 weeks of age, the AL diameter was significantly larger in LG than in SWG, indicating that the adult LG could acquire a stronger immune capacity than SWG (30). Taken together, the spleens of both LG and SWG developed most rapidly from 0 to 10 weeks of age; however, the SWG may have a stronger innate immunity while LG shows a stronger immunomodulatory capacity during post-embryonic development. These data are useful to develop practical management strategies for breed-specific modulation of the immune system during goose postnatal development.

To unravel the crucial genes and pathways commonly and specifically responsible for the spleen development of SWG and LG, we analyzed and compared the genome-wide transcriptomic changes in the spleens of two goose breeds at 0 and 30 weeks of age using RNA-seq technology. The GO enrichment analysis showed that the DEGs identified in spleens of each goose breed between different weeks of age were mostly enriched in the terms related to organ morphogenesis and development, such as nucleus and extracellular space. Similarly, several previous studies have reported that the age has major effects on the development of the goose spleen by regulating cell differentiation (31, 32). Besides, the DEGs identified in spleens of the same age between two goose breeds were mostly enriched in the GO terms related to the structure and ion transport of plasma membrane. Furthermore, results of KEGG enrichment analysis showed that these identified age-related DEGs were mainly enriched in Focal adhesion, Cell cycle, Cytokine-cytokine receptor interaction, Wnt signaling, and ECM-receptor interaction pathways, which have been revealed to regulate the growth and development of immune organs (33, 34). The cell cycle pathway acts as dominate regulator of cell proliferation and mitosis (35), and the neonatal period is characterized by a rapid proliferation and differentiation of lymphocyte (36). Postnatal development of the immune system comprises a series of steps including cell proliferation, differentiation and maturation, among which the proliferation of spleen lymphocyte is the essential one (37). Focal adhesion is the main link between cells and the extracellular matrix and is important for cell proliferation and differentiation (38, 39). Our results together with previous findings suggested that both the Cell cycle and Focal adhesion pathways could play critical roles in regulating the age-dependent goose spleen development. Most breed-related DEGs were shown to be significantly enriched in Neuroactive ligand-receptor interaction, ECM receptor interaction, Wnt signaling, and Calcium signaling pathways. There is evidence that these pathways play important roles in regulating animal immunity (34, 40, 41). Among them, the ECM receptor interaction pathway affects T cell proliferation and apoptosis (42), and in chickens this pathway was shown to be able to regulate the spleen development and immune functions (43), which was consistent with the results obtained in the present study. Previous studies have also shown that calcium regulates various cellular processes by activating or inhibiting cellular signaling pathways and Ca^2+^ regulatory proteins. In chickens, it was recently reported that the calcium signaling pathway is involved in the spleen tumorigenesis (44). In addition, we found that the pathways enriched by the breed-related DEGs differed between these two sampling time points. At 0 week of age, most of the DEGs identified between LG and SWG were enriched in metabolic pathways, which have been previously demonstrated to be important in regulation of poultry embryonic development (45). Thus, we proposed that differential expression of those genes involved in metabolic pathways could result in the breed-related splenic histological and innate immunity differences. However, at 30 weeks of age, most of the DEGs identified between LG and SWG were enriched in the Neuroactive ligand-receptor interaction pathway, which could, at least by part, explain why LG showed a stronger immunity than SWG after being reared for 26 weeks. In support of this, the Neuroactive ligand-receptor interaction pathway has been previously shown to play a role in regulating the different immune responses of different chicken breeds to Salmonella infection (46).

We further performed PPI network analysis to identify crucial genes and pathways during the goose spleen development. By screening the key module from the PPI network constructed with the 690 age-related DEGs, we identified 4 hub genes in the Cell cycle pathway and 3 hub genes in the cell division term. Among them, all of *BUB1*, *BUB1B*, and *TTK* were previously recogized as the crucial genes for spleen development by regualting the proliferation and apoptosis of B and T cells through the Cell cycle pathway (47–49). *BUB1B* is closely related to macrophages and plays an important role in immune regulation (50). Dysregulated expression of *BUB1* and *BUB1B* led to cancer cell proliferation (51, 52). In the pig spleen, *CDK1* was identfied to be a node gene, whose expression levels significantly changed with age (53). Thus, it was inferred that the Cell cycle pathway as well as these hub genes could play crucial roles in regulating the age-dependent goose spleen development. By constructing the PPI network using the 253 breed-related DEGs, we also identfied 3 hub genes in the Neuroactive ligand-receptor interaction pathway, 4 hub genes in the plasma membrane term, and 1 hub gene in the Calcium ion binding pathway. Among them, *RYR2* is abundantly expressed in the splenic sinusoid endothelial cells and plays an important role in filtering out the damaged or aging cells from the blood (54). *GRIA2* and *GRIA4* are involved in regulation of the neuroactive ligand-receptor interactions. The *GRIA2* locus is a key site edited by humans and mice, and biases in this gene editing can lead to severe disease phenotypes (55). Meanwhile, both *GRIA2* and *GRIA4* are involved in regulating expression of genes related to immune response (56). Thus, we inferred that the Neuroactive ligand-receptor interactions pathway as well as these 3 hub genes including *GRIA2*, *GRIA4* and *RYR2* could play cruical roles in regulating the breed-specific goose spleen development. These identified genes and pathways are promising targets for improving goose innate and adaptive immunity by developing new molecular breeding strategies.

## 5 Conclusions

In conclusion, both age and breed are key factors influencing goose spleen development. The spleens of both LG and SWG developed rapidly during post-hatching period of 0-10 weeks. The SWG showed a stronger innate immunity, while LG showed a stronger immunomodulatory capacity during post-hatching development. Comparative transcriptomics analysis further identified a number of significantly enriched pathways and genes regulating the age-and breed-dependent goose spleen development, and among them the Cell cycle and Neuroactive ligand-receptor interaction pathways as well as the associated genes were revealed to play crucial roles. These data provide novel insights into the differences in the splenic development between Chinese and European domestic geese as well the underlying regulatory mechanisms.

## Data availability statement

The transcriptome sequence data has been deposited into the NCBI Sequence Read Archive repository under the accession numbers PRJNA1030932.

## Ethics statement

The animal study was approved by Sichuan Agricultural University Animal Ethical and Welfare Committee. The study was conducted in accordance with the local legislation and institutional requirements.

## Author contributions

SH: Conceptualization, Data curation, Funding acquisition, Methodology, Project administration, Resources, Supervision, Writing – original draft, Writing – review & editing, Formal analysis. YS: Data curation, Formal analysis, Investigation, Validation, Visualization, Writing – original draft, Conceptualization, Methodology. XL: Data curation, Formal analysis, Methodology, Software, Visualization, Writing – original draft. QC: Data curation, Formal analysis, Methodology, Validation, Writing – original draft. BT: Data curation, Methodology, Validation, Writing – original draft, Formal analysis. JC: Data curation, Methodology, Validation, Writing – original draft, Formal analysis. GY: Data curation, Methodology, Validation, Writing – original draft, Formal analysis. HY: Data curation, Methodology, Validation, Writing – original draft, Formal analysis. JuW: Data curation, Resources, Writing – original draft. WW: Data curation, Resources, Writing – review & editing. JH: Data curation, Resources, Writing – review & editing. HH: Data curation, Formal analysis, Methodology, Validation, Writing – review & editing. LL: Data curation, Formal analysis, Methodology, Validation, Writing – review & editing. JiW: Conceptualization, Funding acquisition, Methodology, Resources, Supervision, Validation, Writing – review & editing.

## Supplementary material

Supplementary Figure 1 Principal component analysis and hierarchical clustering analysis of the geese splenic transcriptomes. (A) Principal component analysis of the transcriptomes of 12 samples from LG and SWG. (B) Hierarchical clustering analysis of the total DEGs resulting from LG and SWG comparisons. PCA1, principal component 1; PCA2, principal component 2. LG0, Landes goose at 0 week of age; LG30, Landes goose at 30 weeks of age; SWG0, Sichuan White goose at 0 week of age; and SWG30, Sichuan White goose at 30 weeks of age.
