# Supplementary figures and images for: Comparative transcriptomics analysis identifies crucial genes and pathways during goose spleen development
Source: Front Immunol. 2024 Feb 5;15:1327166. doi: 10.3389/fimmu.2024.1327166 (PMC10875100; doi:10.3389/fimmu.2024.1327166)

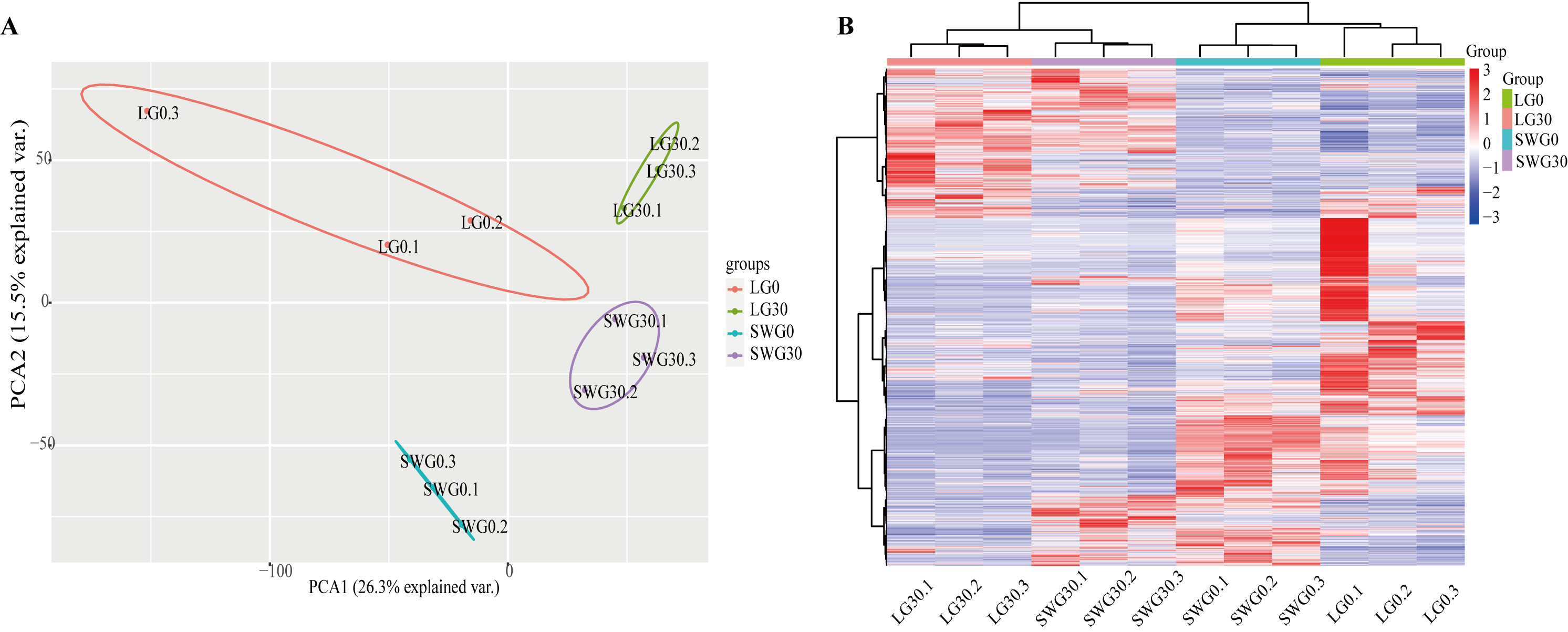

Supplement: Supplementary Figure 1 — Principal component analysis and hierarchical clustering analysis of the geese splenic transcriptomes. (A) Principal component analysis of the transcriptomes of 12 samples from LG and SWG. (B) Hierarchical clustering analysis of the total DEGs resulting from LG and SWG comparisons. PCA1, principal component 1; PCA2, principal component 2. LG0, Landes goose at 0 week of age; LG30, Landes goose at 30 weeks of age; SWG0, Sichuan White goose at 0 week of age; and SWG30, Sichuan White goose at 30 weeks of age. [file Image_1.tif]
