# Supplementary material for: Comparative transcriptomics analysis identifies crucial genes and pathways during goose spleen development
Source: Front Immunol. 2024 Feb 5;15:1327166. doi: 10.3389/fimmu.2024.1327166 (PMC10875100; doi:10.3389/fimmu.2024.1327166)
Supplement: Supplementary file 2 [file Table_1.docx]

Supplementary Table 1. Primer sequences used for quantitative real-time PCR.

| Gene Name | Sequence (5’-3’) | | Product length (bp) |
| --- | --- | --- | --- |
| *BUB1* | F | AGGCAACGCCATTCAAAGTG | 167 |
|  | R | CAAGGTTCCATCAGGCTCCC |  |
| *BUB1B* | F | TATGCCACAGTCATGGGCAG | 87 |
|  | R | GGACGCCTTCCAGAATTCCA |  |
| *CDK1* | F | TGGCAGACTTTGGATTGGCT | 76 |
|  | R | TACCACAGCGTCACGACTTC |  |
| *CENPF* | F | AGTTGGGTTGTGGAGGAGTG | 115 |
|  | R | GGAACTGTCTTTGCTGACGC |  |
| *NDC80* | F | GCAACAGTCAGTATGGCGTG | 163 |
|  | R | ACGGATGGAGACTGTAGGGA |  |
| *NUF2* | F | TCCAGTGTGCCGGGTTAATG | 119 |
|  | R | CACGTCGGGACTCTCTGAAA |  |
| *TTK* | F | AATGCAGCCAGATGTGACGA | 161 |
|  | R | TGCATCCCAATGACCACACG |  |
| *β-ACTIN* | F | TGACAATGGCTCCGGTATGT | 157 |
|  | R | ACCATCACACCCTGATGTCTG |  |
| *GAPDH* | F | AGCAACATCAAGTGGGCAGA | 105 |
|  | R | CACCCATCACGAACATGGGA |  |

F, forward primer; and R, reverse primer.
