# Supplementary material for: Comparative transcriptomics analysis identifies crucial genes and pathways during goose spleen development
Source: Front Immunol. 2024 Feb 5;15:1327166. doi: 10.3389/fimmu.2024.1327166 (PMC10875100; doi:10.3389/fimmu.2024.1327166)
Supplement: Supplementary file 3 [file Table_2.docx]

Supplementary Table 2. Basic information of RNA-seq data.

| Sample | Raw_reads | Clean_reads | Q20(%) | Q30(%) | GC content(%) | Mapping rate (%) |
| --- | --- | --- | --- | --- | --- | --- |
| LG0.1 | 22914904 | 22911093 | 98.12 | 94.05 | 48.99 | 91.23 |
| LG0.2 | 25370861 | 25367977 | 98.19 | 94.32 | 48.69 | 91.39 |
| LG0.3 | 23453948 | 23451667 | 98.14 | 94.16 | 48.86 | 91.95 |
| LG30.1 | 23538980 | 23535529 | 98.18 | 94.30 | 50.92 | 90.20 |
| LG30.2 | 22894038 | 22887452 | 98.18 | 94.33 | 51.31 | 90.00 |
| LG30.3 | 22019483 | 22016991 | 98.29 | 94.65 | 50.58 | 90.22 |
| SWG0.1 | 21803628 | 21801895 | 97.89 | 93.37 | 50.30 | 92.98 |
| SWG0.2 | 25469598 | 25465205 | 98.06 | 93.88 | 49.04 | 93.09 |
| SWG0.3 | 23193286 | 23192095 | 98.29 | 94.60 | 49.6 | 93.38 |
| SWG30.1 | 26588460 | 26586738 | 98.10 | 94.04 | 50.12 | 91.67 |
| SWG30.2 | 22769605 | 22768157 | 98.00 | 93.71 | 49.48 | 91.81 |
| SWG30.3 | 20496153 | 20494500 | 98.20 | 94.35 | 49.78 | 92.43 |

LG0, Landes goose at 0 week of age; LG30, Landes goose at 30 weeks of age; SWG0, Sichuan White goose at 0 week of age; and SWG30, Sichuan White goose at 30 weeks of age.
