# Supplementary material for: Comparative transcriptomics analysis identifies crucial genes and pathways during goose spleen development
Source: Front Immunol. 2024 Feb 5;15:1327166. doi: 10.3389/fimmu.2024.1327166 (PMC10875100; doi:10.3389/fimmu.2024.1327166)
Supplement: Supplementary file 4 [file Table_3.docx]

Supplementary Table 3. Top 10 hub genes identified in PPI network from breed-related DEGs.

| Gene | Gene name | Degree | KEGG pathway/GO term |
| --- | --- | --- | --- |
| *WDR31* | WD repeat domain 31 | 5 | / |
| *GRIP2* | Glutamate receptor interacting protein 2 | 3 | Integral component of membrane |
| *GRIA2* | Glutamate ionotropic receptor AMPA type subunit 2 | 3 | Neuroactive ligand-receptor interaction/Plasma membrane |
| *GRIA4* | Glutamate ionotropic receptor AMPA type subunit 4 | 3 | Neuroactive ligand-receptor interaction/Plasma membrane |
| *RYR2* | Ryanodine receptor 2 | 3 | Calcium signaling pathway/Plasma membrane |
| *RBM20* | RNA binding motif protein 20 | 3 | / |
| *TECTA* | Tectorin alpha | 2 | Plasma membrane |
| *EEFSEC* | Eukaryotic elongation factor, selenocysteine-tRNA specific | 2 | / |
| *FBN2* | Fibrillin 2 | 2 | Calcium ion binding |
| *GRM4* | Glutamate metabotropic receptor 4 | 2 | Neuroactive ligand-receptor interaction |

Degree, the number of nodes directly connected to this node.
